# An educational psychology perspective on health sciences education research

**DOI:** 10.3205/zma001798

**Published:** 2026-01-15

**Authors:** Evelyn Steinberg, Matthias Stadler

**Affiliations:** 1 University of Veterinary Medicine, Vice-rectorate for study affairs, Vienna, Austria; 2 LMU University Hospital, LMU Munich, Institute for Medical Education, Munich, Germany

**Keywords:** educational psychology, health sciences education research, learning, interdisciplinary research

## Abstract

**Background:**

The focus of educational research has shifted from teaching to learning. Psychological theories and methods on student learning are increasingly used in health sciences education (HSE) research. However, applying those theories, methods and practices to HSE poses several challenges.

**Key statements:**

The first challenge relates to theoretical foundations in HSE studies. They are often inadequately described due to differences in writing conventions between psychological and medical disciplines. Moreover, HSE researchers are often trained in medical sciences but not in psychology, leading to potential misconceptions. Interdisciplinary collaboration and more thorough theoretical foundations are essential to overcoming these barriers. The second challenge is implementing effective study designs. HSE research often focuses on improving teaching within individual institutions, but generalizable results require data from multiple institutions. Additionally, HSE researchers without social science training may struggle with study design. Resources for capacity building and systematic educational research are needed, as well as establishing interinstitutional, interdisciplinary networks. The third challenge is the transfer of scientific evidence into educational practice. While it is common sense to provide evidence-based healthcare, less effort is put into providing evidence-based HSE. Quality assurance agencies and ministries can establish regulations pertaining to teacher education to enhance education.

**Conclusion:**

In conclusion, the integration of educational psychology into HSE research poses significant challenges. We need collaborative and interdisciplinary effort, incorporating thorough theoretical frameworks, supportive institutional policies, and effective study designs to address these challenges.

## Zusammenfassung

**Hintergrund:**

Der Fokus der Bildungsforschung hat sich vom Lehren zum Lernen verschoben. Psychologische Theorien und Methoden zum studentischen Lernen finden zunehmend Anwendung in der Erforschung der Aus-, Weiter- und Fortbildung in den Gesundheitsberufen (kurz: Ausbildungsforschung). Dieser Transfer bringt jedoch verschiedene Herausforderungen mit sich.

**Zentrale Aussagen:**

Die erste Herausforderung betrifft die theoretischen Grundlagen der medizinischen Ausbildungsforschung. Aufgrund unterschiedlicher Schreibkonventionen in der Psychologie und in der Medizin werden die theoretischen Grundlagen häufig nicht ausreichend dargestellt. Die zweite Herausforderung ist die Umsetzung effektiver Studiendesigns. Die Ausbildungsforschung konzentriert sich häufig auf die Verbesserung der Lehre in einzelnen Einrichtungen während für die Verallgemeinerung von Ergebnissen eine breitere Datenbasis erforderlich ist. Zudem haben Ausbildungsforscher:innen häufig keine psychologische Ausbildung, was zu Missverständnissen bezüglich theoretischer Grundlagen oder unpräzisen Studiendesigns führen kann. Die dritte Herausforderung ist der Transfer wissenschaftlicher Erkenntnisse in die Lehre. In der Gesundheitsversorgung ist es selbstverständlich, evidenzbasiert zu arbeiten. In der Lehre hingegen spielt die Evidenzbasierung noch keine große Rolle. Um all diesen Herausforderungen zu begegnen sind institutionelle (z.B. Ressourcen, flexible Curricula) und kollaborative (z.B. interdisziplinäre Netzwerke) Bemühungen nötig.

**Schlussfolgerung:**

Die Integration der Bildungspsychologie in die Ausbildungsforschung ist mit erheblichen Herausforderungen verbunden. Eine enge interdisziplinäre Zusammenarbeit und evidenzbasierte Ansätze sind entscheidend, um diese Hürden zu überwinden und nachhaltige Verbesserungen in der Lehre zu ermöglichen.

## Commentary

The shift from teacher-centred higher education to student-centred higher education led to a change from focusing on the science of teaching to focusing on the science of learning. This puts students and their learning center-stage. Educational psychology provides theories, study designs and practices on student learning which are taken up by health sciences education (HSE) research. However, there are some challenges when it comes to transferring educational psychology theories, methods and practices to HSE. This finding applies to various fields of research, such as self-regulated learning or cognitive load, to name just two examples [1], [2].

The first challenge pertains to the theoretical foundation and definition of psychological constructs in HSE studies. They are often inadequately described in the introduction section of HSE manuscripts [2], [3]. This difference may be due to different conventions for writing such manuscripts. The American Psychological Association style emphasizes detailed theoretical foundations in introductions [4] while American Medical Association (AMA) style favors concise introductions emphasizing practical relevance [5]. As a result, psychological theories are hard to fully describe in journals using the AMA’s brief introductions and strict word limits (e.g., self-regulated learning theory depicts complex processes including cognitive, motivational, and emotional aspects). Moreover, HSE researchers are often trained in medical sciences but not in psychology or other social sciences [6]. This makes the transfer process of psychological concepts more difficult and can lead to confusion in terminology and definitions or to misconceptions (e.g. lower cognitive load is always being beneficial to learning [1]). To overcome these challenges, researchers can seek interdisciplinary collaboration and integrate arguments derived from theory into practical arguments. Editors can prioritize theoretical foundations, allow longer manuscripts, and seek theoretically informed reviewer feedback. Reviewers can value interdisciplinary work and engage with diverse theories.

The second challenge is implementing effective study designs [1]. HSE studies often focus on improving teaching at one's own institution, but data from multiple institutions are needed for generalisable results. Educational research designs, like experiments, are difficult to implement due to institutional disinterest, limited resources, inflexible curricula, and time constraints. Additionally, HSE researchers without social science training may struggle with study design. To overcome these challenges, institutions can provide resources, establish a supportive culture as well as more flexible curricula (e.g., a free elective courses, which can be used for educational innovations and research). Capacity building can be achieved through the establishment of a network of interinstitutional and interdisciplinary scientists who receive training and adhere to established methodological standards. If an institution needs help collecting data, they can use their network. Additionally, these researchers may initiate joint projects from the outset. They can collaborate with implementation scientists to develop implementation strategies specific needs of the institutions in question.

The third challenge is the transfer of scientific evidence into educational practice. While it is common sense to provide evidence-based healthcare, less effort is invested in providing evidence-based HSE. Educators are required to manage a range of tasks, including research, clinical, administrative and educational responsibilities [1], which often leaves limited time for their own education. While there are several approaches for addressing these challenges, we highlight one in particular: quality assurance agencies or ministries are well-positioned to establish regulations pertaining to teacher education. For example, the European Association of Establishments for Veterinary Education has recently included such a regulation. 

In conclusion, the transition towards a more student-centered approach in higher education poses significant challenges, particularly in the integration of educational psychology into health sciences education. Addressing these challenges requires a collaborative and interdisciplinary effort, incorporating thorough theoretical frameworks, supportive institutional policies, and effective study designs. Such efforts will not only enhance the generalizability of research findings but also ensure the practical applicability and sustainability of educational innovations. By fostering stronger connections between research, policy, and practice, the field can evolve to meet the demands of both educators and learners in a rapidly changing educational landscape.

## Funding

This research was funded in part by the Austrian Science Fund FWF (P- 33913 G).

## Authors’ ORCIDs

- Evelyn Steinberg: [0000-0001-5464-3675]
- Matthias Stadler: [0000-0001-8241-8723]

## Competing interests

The authors declare that they have no competing interests. 
